# Room-temperature Domain-epitaxy of Copper Iodide Thin Films for Transparent CuI/ZnO Heterojunctions with High Rectification Ratios Larger than 10^9^

**DOI:** 10.1038/srep21937

**Published:** 2016-02-26

**Authors:** Chang Yang, Max Kneiß, Friedrich-Leonhard Schein, Michael Lorenz, Marius Grundmann

**Affiliations:** 1 Institut für Experimentelle Physik II, Universität Leipzig, Leipzig, 04103, Germany

## Abstract

CuI is a p-type transparent conductive semiconductor with unique optoelectronic properties, including wide band gap (3.1 eV), high hole mobility (>40 cm^2 ^V^−1 ^s^−1^ in bulk), and large room-temperature exciton binding energy (62 meV). The difficulty in epitaxy of CuI is the main obstacle for its application in advanced solid-state electronic devices. Herein, room-temperature heteroepitaxial growth of CuI on various substrates with well-defined in-plane epitaxial relations is realized by reactive sputtering technique. In such heteroepitaxial growth the formation of rotation domains is observed and hereby systematically investigated in accordance with existing theoretical study of domain-epitaxy. The controllable epitaxy of CuI thin films allows for the combination of p-type CuI with suitable n-type semiconductors with the purpose to fabricate epitaxial thin film heterojunctions. Such heterostructures have superior properties to structures without or with weakly ordered in-plane orientation. The obtained epitaxial thin film heterojunction of p-CuI(111)/n-ZnO(00.1) exhibits a high rectification up to 2 × 10^9^ (±2 V), a 100-fold improvement compared to diodes with disordered interfaces. Also a low saturation current density down to 5 × 10^−9 ^Acm^−2^ is formed. These results prove the great potential of epitaxial CuI as a promising p-type optoelectronic material.

Over the last decades transparent conducting materials (TCMs) had a dramatic increase in interest in the field of optoelectronics. Fully-transparent devices gave rise to a novel concept of transparent electronics^1^. To realize advanced active devices, it is necessary to have materials that can be electron- *and* hole-conducting. Except for the group-III nitrides^2^, however, most TCMs are unipolar n-type semiconductors such as ZnO^3^, GaInZnO^4^. Thus bipolar devices will largely depend on heterostructures from n-type and p-type materials. Therefore, it is of great importance to explore new p-type TCMs with superior optical and electrical characteristics which are compatible to the commonly used n-type TCMs.

Copper iodide in the zincblende ground-state phase (*γ*-CuI) has p-type conductivity with a high Hall mobility (>40 cm^2 ^V^−1 ^s^−1^ in bulk)^5^,^6^, a wide band gap (3.1 eV) with a direct band structure, and a large exciton binding energy (62 meV)^6^. These advantageous properties make it one of the most promising p-type TCMs. Actually, it is historically the first TCM found by Bädeker as early as 1907^7^,^8^. CuI has been successfully applied in scintillators^9^, organic electronics^10^,^11^, and bipolar diodes^12^,^13^,^14^. The development of high quality bipolar diodes opens up the application of CuI in transparent electronics.

Polycrystalline heterojunctions of p-type CuI are reported combining with various n-type semiconductors, such as n-Si, n-TiO_2_ and n-ZnO. So far, the highest rectification of diodes involving CuI as p-side is obtained for p-CuI/n-ZnO heterojunctions. In refs 14,15, CuI thin films were obtained from vapor iodization of copper films or from thermal evaporation of CuI powders, exhibiting an already fairly high rectification (current on/off-ratio) of about 4 × 10^7^ (±2 V). However, the device performance was dominantly limited by the poor crystallinity of CuI.

In the fabrication of advanced solid-state electronic and photonic devices, epitaxial growth and the control, reduction and possibly avoidance of defects in thin film heterostructures are usually the key considerations. From this point of view, epitaxy of *γ*-CuI and combination with suitable n-type semiconductors is a critical issue.

Up to date, it is still a challenge to grow epitaxial thin films of *γ*-CuI due to the lack of lattice-matched substrates. Rather than for semiconductors, only for single-crystalline Cu(111) or NaCl(001) substrates heteroepitaxy of *γ*-CuI was reported^16^,^17^. A study in thermal evaporation of *γ*-CuI on ZnO epilayer revealed the possible existence of epitaxial crystallites within the polycrystalline CuI matrix^15^, indicating the opportunity of epitaxial growth of *γ*-CuI at appropriate growth conditions. However, thermal evaporation is not a very advantageous method for epitaxial growth due to the uncontrollable and high deposition rate which is commonly over 1 nm/s. Advanced thin film growth techniques, such as sputtering, molecular beam epitaxy (MBE), metal-organic vapor phase epitaxy (MOVPE) or pulsed laser deposition (PLD), seem more suitable for well-controlled epitaxy of CuI.

In the presence of lattice mismatch, domain epitaxy allows for a variety of advanced matching conditions^18^. The minimum energy lattice-matching condition involves rotation of the epilayer crystal lattices. In heteroepitaxy, rotation domains are a fundamental consequence of the mismatch of rotational symmetry across the heterointerface. The unified theory of formation of rotation domains was described elsewhere^19^,^20^. With the guidance of this theory, we systematically investigate the epitaxy of *γ*-CuI thin films fabricated by reactive sputtering at room temperature on various substrates with the purpose to achieve epitaxy with a well-defined in-plane epitaxial relation. We generally expect such heterostructures to have superior properties to structures without or with weakly ordered in-plane orientation^2^,^7^. Here we report such advanced epitaxial thin film heterojunctions of p-CuI(111)/n-ZnO(00.1) with a high rectification of 2 × 10^9^ (±2 V) of the diodes.

## Results and Discussion

### Epitaxy

**Figure 1:**
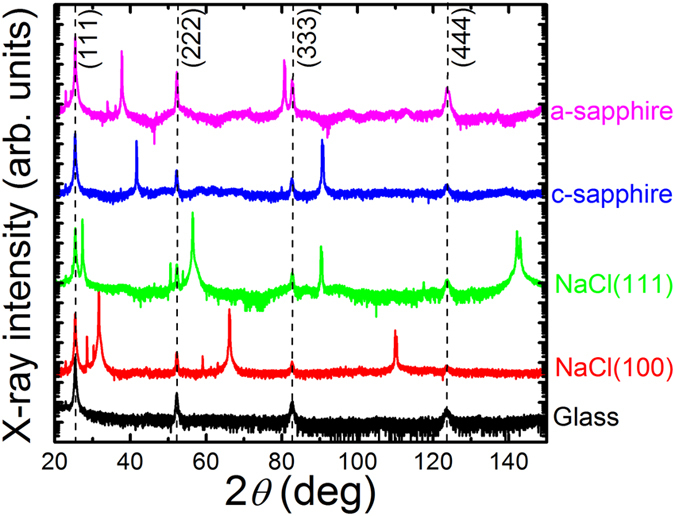
X-ray diffraction (XRD) 2*θ*-*ω*-scans of CuI thin films grown on the indicated NaCl and sapphire single-crystal substrates and glass. Dashed lines are guide to the eyes corresponding to the partly weak diffraction peaks of the CuI thin films. The additional peaks in the patterns can be assigned to the corresponding substrate materials including Kβ and Lα spectral lines.

Figure 1 shows X-ray diffraction (XRD) 2*θ*-*ω*-scans of CuI thin films grown on NaCl and sapphire single-crystal substrates with different crystal orientations and glass. All XRD patterns exhibit peaks corresponding to the (111), (222), (333) and (444) planes of CuI in zincblende structure, suggesting the growth of *γ*-CuI along the [111]-direction on all substrates. This result agrees with the reported growth nature of *γ*-CuI^21^. The additional XRD peaks that are seen in the patterns can be assigned to the single-crystal substrates.

The structural properties of selected substrates and bulk *γ*-CuI are compiled in Table 1. It is obvious that large lattice mismatch (along a-axis) exists for most substrates, which tends to induce a significant density of dislocations in the *γ*-CuI epilayer and thereby hinder the conventional epitaxial growth. In this case, domain epitaxy is favored for unconventional in-plane lattice matching. Rotation domains occur, when the rotational symmetry of the substrate surface and epilayer are different. The substrate surface and epilayer rotational symmetries are denoted as *C*_*n*_ and *C*_*m*_, respectively. The minimum number of rotation domains *N*_RD_ is given by^19^


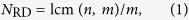


where lcm denotes the least common multiple. In this work, the as-deposited *γ*-CuI thin films exhibit (111)-orientation along the surface normal for all substrates, thus setting the 3-fold symmetry (*m* = 3) of the epilayer. Therefore, the predicted *N*_*RD*_ depends fundamentally on the *C*_*n*_ symmetry of the substrate.

**Figure 2:**
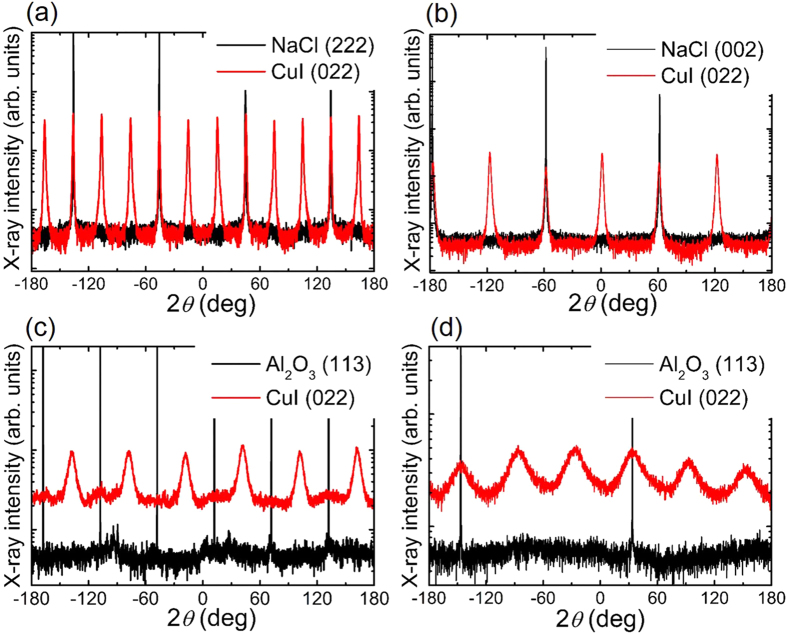
XRD *ϕ*-scans around the surface normal of CuI thin films grown on (**a**) NaCl(001), (**b**) NaCl(111), (**c**) c-sapphire and (**d**) a-sapphire substrates. The sharp black peaks stem from the indicated substrate materials.

The rotational symmetries of the substrates used in this study are *n* = 2, 3, 4 and 6, namely for a-sapphire [Al_2_O_3_(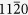)], NaCl(111), NaCl(001), c-sapphire [Al_2_O_3_(0001)], respectively. The azimuthal (in-plane) orientation of the *γ*-CuI epilayers with respect to the substrate was characterized by XRD *ϕ*-scans around the surface normal. As shown in Fig. 2, well-defined CuI(022) peaks periodically appear for all these samples, demonstrating the successful heteroepitaxial growth of *γ*-CuI on selected single-crystals. For each single-crystal substrate, the number of diffraction peaks observed in *ϕ*-scan corresponds well to its symmetry. For example, the *ϕ*-scan of (222) plane of the NaCl(001) substrate (*n* = 4) shows four peaks with a separation of 90° due to the 4-fold rotational symmetry of rocksalt in (001) direction. Considering the 3-fold rotational symmetry of the (111)-oriented CuI epilayer, a single domain exhibits three peaks with *ϕ*-scan for the asymmetric (022) peak. However, there are twelve peaks observed by *ϕ*-scan of CuI(022), indicating four rotation domains (*N*_RD_ = 4) rotated by 30° (or 90°) against each other. Differently, for other CuI films on substrates of Al_2_O_3_(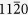) (*n* = 2), NaCl(111) (*n* = 3) and Al_2_O_3_(0001) (*n* = 6), six CuI(022) peaks in *ϕ*-scans indicate the presence of two rotation domains (*N*_RD_ = 2) with relative azimuthal orientation of 60°. This result agrees with the minimum number of rotation domains *N*_RD_ deduced by Eq. 1. We note that on NaCl(111) (*n* = 3), the *N*_RD_ has a theoretical minimum value of one but an experimental value of two. It is owing to the formation of mirror twinned CuI(111) domains. This phenomenon is similar to the growth of TiN(111) on Si(111), where fully twinned material was obtained^22^.

**Figure 3:**
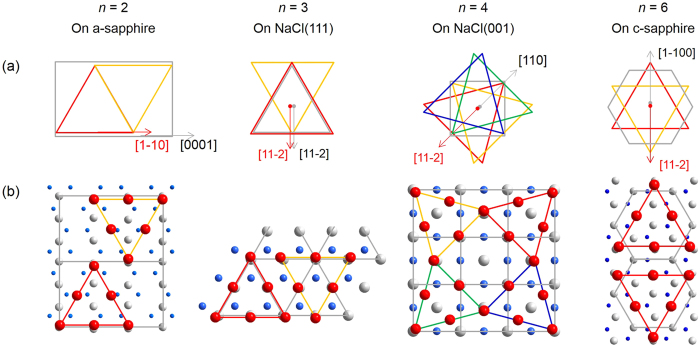
(**a**) Schematic alignment and (**b**) atomic configurations of CuI crystallites epitaxially grown on NaCl and sapphire substrates. In (**b**) blue sphere: cation of substrate material; grey sphere: anion of substrate material; red sphere: iodine ions of CuI epilayer.

According to the angular peak positions in XRD *ϕ*-scans, as depicted schematically in Fig. 3(a), the heteroepitaxial relationships can be deduced as CuI[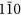](111) || Al_2_O_3_[0001](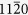), CuI<
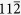
>(111) || NaCl[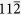](111), CuI<
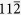
>(111) || NaCl[110](001), and CuI<
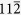
>(111) || Al_2_O_3_[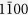](0001). The formation of these different rotation domains can be explained using atomic configurations as sketched in Fig. 3(b). The material combinations for *γ*-CuI epilayer on selected substrates in all conditions of rotational symmetry are summarized in Table 2.

These results show that our sputter deposition from metallic Cu target in ioidine/argon atmosphere is suitable for achieving epitaxial layers. Also we further corroborate previous theoretical studies on rotation domains^19^,^20^. It reveals the typical conditions in heteroepitaxy of CuI thin film on various substrates, paving the way to fabricate epitaxial multilayers for advanced heterojunctions. For a functional heterostructure we investigate in the following epitaxy on ZnO which serves as n-type contact in a bipolar diode.

**Figure 4:**
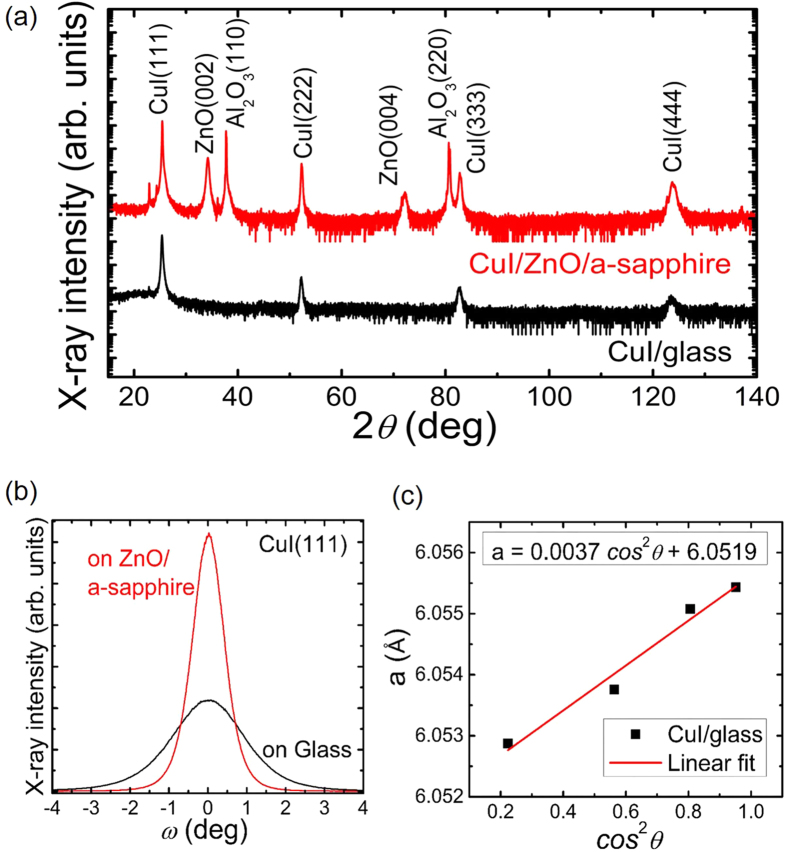
(**a**) XRD 2θ-ω-scans of CuI/ZnO/a-sapphire and CuI/glass, (**b**) XRD *ω*-scans of CuI(111) on ZnO/a-sapphire and glass, respectively, and (**c**) lattice constant *a* of CuI grown on glass determined from a cos^2^*θ*-extrapolation of the peak positions in the 2*θ*-*ω*-scan to *θ* = 90°. In (**c**) the precise lattice constant is obtained from the interception of the linear fit of the data with y-axis at cos^s^ *θ* = 0.

Figure 4(a) shows the XRD 2*θ*-*ω* patterns of CuI/ZnO multilayers on a-sapphire and (amorphous) glass substrates, respectively. The PLD of such ZnO epilayer on sapphire has been described previously and exhibits c-plane orientation, i.e. the out-of-plane epitaxial relation ZnO[0001] || Al_2_O_3_[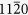]^23^. On both ZnO(0001) and glass the CuI exhibits (111) orientation. As shown in Fig. 4(b), the full width at half maximum (FWHM) of CuI(111) rocking curve is significantly smaller on ZnO than on glass.

From the peak positions in the 2*θ*-*ω*-scans, the out-of-plane lattice constant of CuI thin film can be calculated by applying the Bragg equation for cubic crystals. To minimize the height error of the goniometer, an extrapolation with the function *a* = *f* (cos^2^ *θ*) is performed. The linear fit provides the lattice constant at cos^2^ *θ* = 0, i.e. at *θ* = 90°. Figure 4(c) shows the extrapolation of the lattice constant of CuI/glass of *a* = (6.052 ± 0.001) Å, which is very close to the bulk value. The lattice constants of all the samples on different substrates are treated with the same fitting process, which can be found in Supplementary Table S1. There is no clear dependence of lattice constants on substrates, probably due to the low growth temperature (room temperature) under which the lattice strains release easily. However, comparing with the polycrystalline CuI grown on glass, the samples on single-crystalline substrates exhibited generally small FWHM values of CuI(111) peaks in rocking curves, indicating the reduced tilt mosaicity of CuI thin films by epitaxy.

**Figure 5:**
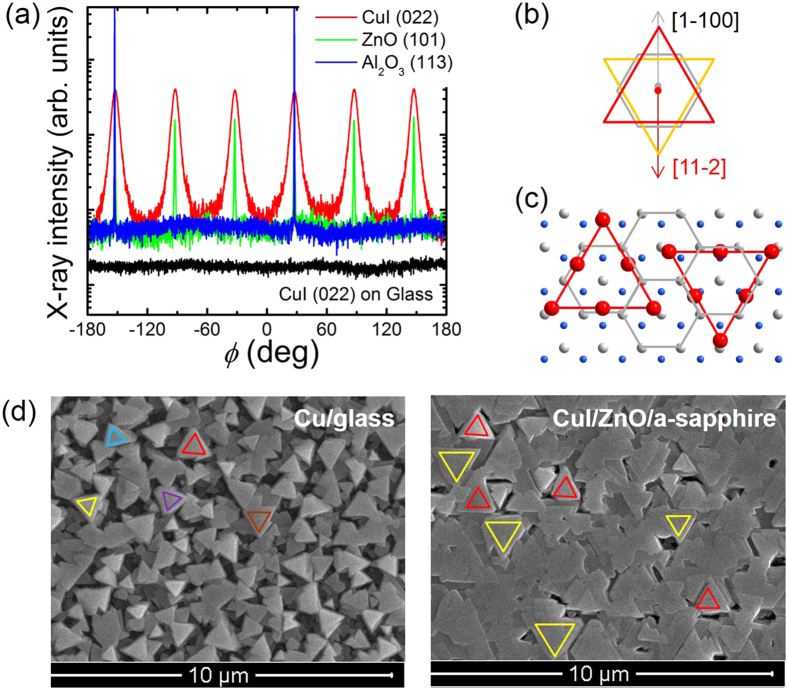
(**a**) XRD *ϕ*-scans of sample CuI/ZnO/a-sapphire and CuI/glass, (**b**) schematic alignment and (**c**) atomic configurations of CuI lattices on c-plane ZnO surface, and (**d**) SEM images of CuI thin films on glass and ZnO/a-sapphire respectively. In (**d**), triangular grains have been marked according to their orientation. Only two different orientations of the triangles have been found for CuI on ZnO/a-sapphire. Examples for each group are marked by colored triangles in red and yellow.

While the different rocking curve widths of the CuI/ZnO and CuI/glass thin films suggest a quantitatively better crystallinity of the CuI/ZnO layer, a major qualitative difference between the two systems is revealed in the XRD *ϕ*-scans as depicted in Fig. 5(a). The X-ray scattering intensity in a *ϕ*-scan for CuI/glass is constant over the angle and thus, as expected for deposition on an amorphous substrate, no preferred azimuthal orientation of CuI grains is present. In contrary, clearly defined peaks are found for the CuI/ZnO system. The c-axis-oriented ZnO epilayer offers a 6-fold symmetry similar to c-sapphire (*n* = 6), resulting in six peaks of ZnO(10.1). Six CuI(022) peaks separated by 60° were observed, indicating the heteroepitaxy of CuI on ZnO. This corresponds to the number of minimum *N*_RD_ = 2 determined from Eq. 1, which is similar to the case of epitaxy on c-sapphire. The epitaxial relationship of CuI<
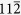
>(111) || ZnO[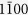](0001) can be deduced in Fig. 5(b), and the corresponding atomic configurations are shown in Fig. 5(c).

The surface morphology of the CuI thin films is investigated with scanning electron microscopy (SEM). As shown in Fig. 5(d), the SEM image on the left clearly reveals triangularly shaped grains on glass substrate, which confirms the (111)-oriented *γ*-CuI according to XRD patterns. However, rather than a continuous thin film it was comprised of loosely compacted *μ*m-sized crystallites. The random orientation of these grains indicates a thin film without in-plane epitaxial order and with poor quality (non-continuous substrate covering and arbitrary grain boundaries). On the other hand, the CuI epilayer grown on the ZnO surface (see SEM image on the right in Fig. 5(d)) exhibits a compact thin film with a larger grain size than that of the CuI/glass thin film. On closer inspection each grain of triangular shape can be assigned to one of the two groups marked by the different colors in Fig. 5(d) according to their orientation. On comparison of the triangles of two rotational domains, they are rotated with respect to each other with an angle of 180° (or 60°). This confirms the conclusion from the XRD *ϕ*-scan that the CuI thin film grows epitaxially on the c-plane ZnO surface. Compared to the polycrystalline sample, this epitaxial thin film of CuI has a significantly higher crystal quality which is favorable for device fabrication.

### Diodes

**Figure 6:**
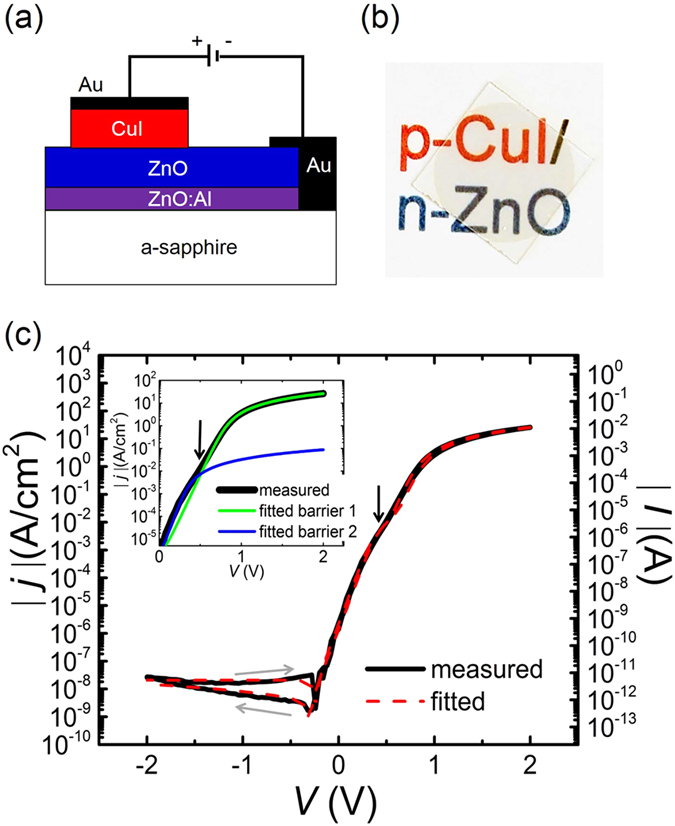
(**a**) Schematic of the CuI/ZnO diode structure, (**b**) photograph of CuI/ZnO bilayer on a-sapphire substrate, and (**c**) Current density vs. voltage characteristic of CuI/ZnO diode. The inset depicts the characteristics of the fitted multiple barriers near the kink region. Black arrow: kink at ~0.6 V probably related to inhomogeneous diode properties. Grey arrow: voltage sweeping direction.

Based on these results, bipolar heterodiodes were fabricated from epitaxial p-CuI/n-ZnO on a-sapphire heterostructures. The schematic of the fabricated heterodiodes is shown in Fig. 6(a). The sputtered gold contacts on CuI were checked to be Ohmic. In order to achieve a good Ohmic contact of the obtained n-ZnO layer, a ZnO:Al current spreading layer is introduced as a back Ohmic contact with gold to reduce the series resistance^23^,^24^,^25^. The diode characteristics of current density *j* vs. voltage *V* can be descripted by the equation


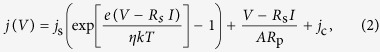


where *R*_s_ (*R*_p_) denotes the series (parallel) resistance, *η* the ideality factor, *T* the absolute temperature, *k* Boltzmann’s constant, *A* the junction area, *j*_s_ the saturation current density and *j*_c_ an offset current to take capacitive effects into account.

Figure 6(c) shows the *j*-*V* characteristic of one of the best specimen of such CuI/ZnO diodes (with contact area A = 2.25 × 10^−4 ^cm^2^). However, in forward direction, two distinct regions can be detected where *j* depends exponentially on *V*. This behavior could be attributed to the lateral variation of the CuI layer within the contact area according to the multi-contact theory^26^. The inset of Fig. 6(c) depicts the characteristics of the two barriers which result in a kink of measured *j*–*V* curve at ~0.6 V. Hence the diode characteristics are numerically fitted with multi-contact model as the dashed red line in Fig. 6(c). Below 0.6 V the ideality factor *η* is ~1.7 and the saturation current density *j*_*s*_ is ~5 × 10^−9 ^Acm^−2^. While above 0.6 V the lateral variation of the CuI layer within the contact area results in a larger *η* value of ~2.2. Further fit parameters can be found in Supplementary Table S2.

Our epitaxial CuI/ZnO diodes exhibit generally high rectifications in the range from 10^7^ to 10^9^ at ±2 V. The best rectification ratio (see Fig. 6(c)) reaches up to ~2 × 10^9^ (±2 V), which is nearly two orders of magnitude higher than that of polycrystalline diodes^15^. As far as we know, no report in the literature on crystalline diodes involving ZnO or other n-type wide bandgap oxide semiconductors exceeds a rectification of 10^3^ except a p-6H-SiC/n-ZnO diode reaches 1.3 × 10^4^ at ±8 V^27^, and a p-NiO_x_/n-TiO_x_ diode reaches 6.7 × 10^5^ at ±2 V^28^. Hence our epitaxial p-CuI/n-ZnO diode compares favorably with other diode from n-type oxide semiconductors and amorphous p-type oxides (ZnCo_2_O_4_ and NiO)^25^,^29^,^30^ and is additionally fully transparent (see Fig. 6(b)). This result demonstrates the advantages and high potential of CuI in application of transparent electronics.

## Conclusion

In summary, heteroepitaxial growth of CuI on various substrates at room temperature was realized. The formation of rotation domains was systematically investigated. The epitaxial relationships of CuI[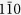](111) || Al_2_O_3_[0001](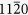), CuI<
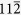
>(111) || NaCl[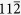](111), CuI<
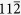
>(111) || NaCl[110](001), CuI<
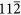
>(111) || Al_2_O_3_[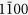](0001), and CuI<
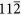
>(111) || ZnO[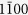](0001) were established. These results enable the controllable domain epitaxy of CuI thin films on various substrates. Based on the epitaxial p-CuI/n-ZnO heterostructure, bipolar diodes were demonstrated with a rectification ratio of ~2 × 10^9^ at ±2 V, which is nearly two orders of magnitude higher than that made of polycrystalline CuI. This value presents by far the highest rectification of crystalline diodes involving oxide semiconductors. The low growth temperature (room temperature) and the use of sputtering technique make it a facile way to produce large area CuI epilayers for applications in transparent electronics.

## Methods

The CuI films were deposited by DC sputtering in a dynamically pumped chamber with a base pressure of ~1 × 10^−5^ millibar. A high purity (99.999%) copper disk was used as the sputtering target. For the deposition of CuI thin films, iodine vapor was introduced by a needle valve connected with a heated iodine source. This iodine source was a stainless steel tube filled with iodine particles, which was kept at ~180 °C to sustain a sufficient iodine vapor pressure. The bearing of the turbomolecular pump is protected by nitrogen gas. The throttle valve between the chamber and turbomolecular pump was partially closed for adjusting the iodine partial pressure to ~1 × 10^−3^ millibar. Argon was introduced to set the total pressure to 0.02 millibar.

Presputtering was conducted with power of 30 W for 20 min with the shutter closed. For investigating various epitaxial relationships, substrates with different rotational symmetry were selected including NaCl(001), NaCl(111), a-plane and c-plane sapphire single-crystals, as well as amorphous Corning 1737 glass for a comparison. The CuI samples were sputtered at 30 W at room temperature. The typical thickness of CuI thin film was estimated as ~100 nm with a Dektak profilometer. Heterojunction of CuI with ZnO was subsequently fabricated. The 40 nm thin ZnO:Al (1 wt% Al_2_O_3_) current spreading layer (electrical resistivity *ρ* = 1 × 10^−3 ^Ωcm) and the 100 nm thin ZnO film were grown successively by PLD at 650 °C and 0.016 millibar oxygen partial pressure on an a-plane sapphire substrate^23^.

Investigations of the epitaxial relationship were performed with a Philips X’Pert x-ray diffractometer equipped with a Bragg-Brentano powder goniometer using divergent/focusing slit optics and Cu K*α* radiation. SEM was done using a FEI NOVA Nanolab 200. The *I*-*V* measurement was performed with a Süss wafer prober and an Agilent 4155C Semiconductor Parameter Analyzer.

## Additional Information

**How to cite this article**: Yang, C. *et al.* Room-temperature Domain-epitaxy of Copper Iodide Thin Films for Transparent CuI/ZnO Heterojunctions with High Rectification Ratios Larger than 10^9^. *Sci. Rep.* **6**, 21937; doi: 10.1038/srep21937 (2016).

## Supplementary Material

Supplementary Information

## Figures and Tables

**Table 1:** Structural properties of bulk *γ*-CuI and the used single-crystalline substrates.

Material	Space group	Crystalstructure	Lattice constant(Å)	a-axis mismatchwith *γ*-CuI
*γ*-CuI	Fm-3m (225)	cubic	a = 6.05	–
NaCl	Fm-3m (225)	cubic	a = 5.64	−7.3%
Al_2_O_3_	R-3c (167)	rhombohedral	a = 4.76, c = 12.99	−27.1%
ZnO	P63mc (186)	hexagonal	a = 3.24, c = 5.19	−86.7%

**Table 2:** Material combinations for (111)-oriented *γ*-CuI epilayer (E) on various substrates (S) with different symmetry *C*_*n*_.

Symmetry *C*_*n*_	Substrate	Out-of-plane E || Sepitaxial relation	In-plane E || Sepitaxial relation	Theoretical Minimum*N*_RD_	Experimental*N*_RD_
∞	glass	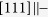	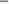	∞	>>> 1
2	Al_2_O_3_( 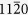 )	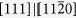	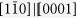	2	2
3	NaCl(111)	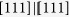	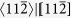	1	2
4	NaCl(001)	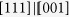	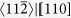	4	4
6	Al_2_O_3_(0001)	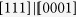	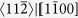	2	2
6	ZnO(0001)	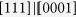	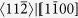	2	2

The ZnO(0001) surface is provided by a c-axis-oriented ZnO epilayer grown on a-sapphire.
