# Supplementary material for: Room-temperature Domain-epitaxy of Copper Iodide Thin Films for Transparent CuI/ZnO Heterojunctions with High Rectification Ratios Larger than 109
Source: Sci Rep. 2016 Feb 26;6:21937. doi: 10.1038/srep21937 (PMC4768143; doi:10.1038/srep21937)
Supplement: Supplementary Information [file srep21937-s1.pdf]

Room-temperature Domain-epitaxy of Copper Iodide Thin Films for Transparent CuI/ZnO Heterojunctions with High Rectification Ratios Larger than 10^9

Chang Yang*, Max Kneiß, Friedrich-Leonhard Schein, Michael Lorenz, and Marius Grundmann

Institut für Experimentelle Physik II, Universität Leipzig, Leipzig, 04103, Germany
*yangchangyc@gmail.com

Supplementary Table S1. Crystallinity of γ-CuI films grown on various substrates evaluated by FWHM of CuI(111) peaks by XRD ω-scans. The lattice constant along the growth direction is determined from the $cos^2\theta$-extrapolation at θ = 90 °of the peak positions in XRD 2θ-ω-scans of the CuI thin films. The ZnO(0001) surface is provided by a PLD grown c-axis-oriented ZnO epilayer on a-sapphire.

Substrate	FWHM of CuI(111) (°)	Lattice constant (Å)	In-plane lattice mismatch (%)
glass	2.276	6.0519(3)	—
Al_2O_3(0001)	1.539	6.0512(2)	10.3
Al_2O_3(11$\bar{2}$0)	1.041	6.0494(2)	1.1 ~ 9.3
NaCl(001)	0.988	6.0493(8)	3.8
NaCl(111)	0.964	6.0491(8)	-7.1
ZnO(0001)	0.967	6.0506(1)	13.0

Supplementary Table S2. Diode parameters ($A = 2.25\times10^{-4}$ cm^2, T = 300 K) calculated using multi-contact model for the *j*-*V* characteristic shown in Fig. 6(c).

Sweeping direction	Barrier 1 ($V > 0.6$ V)			Barrier 2 ($V < 0.6$ V)			R_p (Ω)
	η	j_s (Acm^{-2})	R_s (Ω)	η	j_s (Acm^{-2})	R_s (Ω)	
Forward	2.22	2.07×10^{-8}	174.70	1.68	6.13×10^{-9}	8.11×10^{4}	3.40×10^{12}
Reverse	2.16	5.07×10^{-9}	177.17	1.72	5.01×10^{-9}	4.40×10^{4}	8.04×10^{11}
